# Sweat gland nerve fiber density and association with sudomotor function, symptoms, and risk factors in adolescents with type 1 diabetes

**DOI:** 10.1007/s10286-023-00973-7

**Published:** 2023-09-08

**Authors:** Vinni Faber Rasmussen, Ann Schmeichel, Mathilde Thrysøe, Jens Randel Nyengaard, Ann-Margrethe Rønholt Christensen, Esben Thyssen Vestergaard, Kurt Kristensen, Astrid Juhl Terkelsen, Páll Karlsson, Wolfgang Singer

**Affiliations:** 1 Danish Pain Research Center, Department of Clinical Medicine, Aarhus University, Aarhus, Denmark; 2 Department of Pediatrics and Adolescents, Randers Regional Hospital, Randers, Denmark; 3 Steno Diabetes Center Aarhus, Aarhus University Hospital, Aarhus, Denmark; 4 Department of Neurology, Mayo Clinic, Rochester, MN USA; 5 Core Center for Molecular Morphology, Section for Stereology and Microscopy, Department of Clinical Medicine, Aarhus University, Aarhus, Denmark; 6 Department of Pathology, Aarhus University Hospital, Aarhus, Denmark; 7 Steno Diabetes Center North Denmark, Aalborg, Denmark; 8 Department of Pediatrics and Adolescents, Aalborg University Hospital, Aalborg, Denmark; 9 Department of Pediatrics and Adolescents, Aarhus University Hospital, Aarhus, Denmark; 10 Department of Neurology, Aarhus University Hospital, Aarhus, Denmark

**Keywords:** Sweat gland nerve fiber density, Type 1 diabetes, Adolescents, Quantitative sudomotor axon reflex test, Neuropathy

## Abstract

**Purpose:**

To quantify sweat gland nerve fiber density in adolescents with diabetes. Additionally, to investigate associations between sudomotor innervation, sweat responses, and possible risk factors for sudomotor neuropathy.

**Methods:**

Cross-sectional study where 60 adolescents with type 1 diabetes (duration > 5 years) and 23 control subjects were included. Clinical data, quantitative sudomotor axon reflex test, and skin biopsies were obtained. Skin tissue was immunostained and imaged by confocal microscopy. Quantification of the sweat gland volume and three-dimensional reconstruction of the nerve fibers was performed using a design-unbiased technique.

**Results:**

Adolescents with diabetes had a significant reduction of maximum and mean values of nerve fiber length and nerve fiber density in sweat glands compared to controls (*p* values < 0.05). No association between nerve fiber density and sweat responses was found (*p* = 0.21). In cases with reduced sweat gland nerve fiber length, nerve fiber density, and volume, the sweat response was reduced or absent. Height, systolic blood pressure, time in hypoglycemia, and total daily and basal/total insulin dose were positively correlated to sweat response, while low-density lipoprotein, and HbA1c were negatively correlated with sweat response (*p* values < 0.05). Other microvascular complications and high cholesterol levels increased the relative risk for reduced sweat gland nerve fiber density.

**Conclusion:**

Our findings of reduced sweat gland innervation in a selected group of adolescents add new knowledge about the structural changes that occur in autonomic nerves due to diabetes. Evaluating both the sweat gland innervation and sweat gland volume was important for understanding the association with sweat responses. Further research is needed to understand its clinical relevance.

**Supplementary Information:**

The online version contains supplementary material available at 10.1007/s10286-023-00973-7.

## Introduction

The prevalence of autonomic neuropathy among adolescents with type 1 diabetes (T1D) ranges widely, with estimates ranging from 4 to 75%, based primarily on cardiovascular autonomic function tests [1, 2]. However, diabetic autonomic dysfunction can impact multiple organs, including the cardiovascular, urogenital, gastrointestinal, pupillomotor, thermoregulatory, and sudomotor systems. Among these, sudomotor dysfunction is a common and early aspect of diabetic autonomic neuropathy [3] and typically causes decreased sweating in the distal parts of the body. Abnormal autonomic sudomotor innervation can lead to hypohidrosis, anhidrosis, and hyperhidrosis [4], with the last being a compensatory phenomenon [3]. Research by Gibbons et al. revealed a significant reduction in sudomotor innervation in adults with diabetes (*n* = 36, mean diabetes duration between 4.6 and 13 years depending on sex and diabetes type) compared with healthy controls. The study group also suggested that sweat gland nerve fiber quantification can be done rapidly and reproducibly [5] and that sweat gland nerve fiber density (SGNFD) can differentiate between adults with mild diabetic neuropathy and healthy controls [6]. Additionally, Luo et al. found lower sweat gland innervation indices in adults with type 2 diabetes (*n* = 35) [7], and Liu et al. observed a decrease in SGNFD over a 1-year period without any progression in intraepidermal nerve fiber density (IENFD) [8].

Quantifying SGNFD using 3D reconstruction of sudomotor innervation has shown great potential for diagnosis with high reproducibility [9]. The method provides an evaluation of structural nerve damage, while the quantitative sudomotor axon reflex test (QSART) assesses the functional sweat response after nerve stimulation. QSART has been validated as an effective method in autonomic testing [10]; however, it has not yet been applied to adolescents with T1D.

The aim of this study was to determine SGNFD in adolescents with T1D and to investigate the association between SGNFD, sweat response obtained by QSART, and potential risk factors for abnormal sudomotor function and sweat gland nerve fiber loss.

## Methods

The study was a cross-sectional study.

### Study population

Adolescents with T1D were recruited among patients who were visiting a hospital or diabetes center for regular diabetes management between August 2020 and December 2021. The participants were recruited continuously, and participation was voluntary. The facilities that were included in the study in Denmark included the Departments of Pediatrics and Adolescent Medicine at Randers Regional Hospital, Aarhus and Aalborg University Hospital, and the Steno Diabetes Centers in Aarhus and North Denmark. Inclusion criteria were age 15 to 18 years, T1D, and diabetes duration of more than 5 years. The International Society for Pediatric and Adolescent Diabetes (ISPAD) criteria for the diagnosis of diabetes were used [11]. The presence of autoimmune markers, such as specific antibodies (e.g., anti-GAD, anti-IA2), provided additional evidence to support the diagnosis of T1D and differentiate it from other forms of diabetes [11]. Additionally, an age-matched healthy control group was enrolled.

Participants were excluded if they were on medications or had other diseases (e.g., B12 deficiency) which could impact the central or peripheral nervous system. Healthy age-matched controls were recruited from boarding and secondary schools. Study participants were from the T1DANES cohort (T1D = type 1 diabetes, D = Danish population, A = adolescents, N = neuropathy, ES = explanatory study). All participants underwent additional neurological examinations not described in this paper [12, 13].

Informed oral and written consent was obtained from each participant and the accompanying parents. All procedures in the study protocol were approved by the Danish Ethics Committee (Project ID M-2019-211-19) and Legal Office, Central Denmark Region (1-16-02-42-21).

### Clinical and biochemical data collection

Clinical and biochemical data were extracted from the medical records, and any missing data were collected on the test day. Healthy participants underwent a fasting blood sample taken for analysis of hemoglobin A1c and lipid profile (total cholesterol, low-density and high-density lipoprotein, and triglycerides) on the test day.

Weight, height, hip, and waist measurements were obtained, and body mass index (BMI) was calculated as weight divided by height squared. Blood pressure and heart rate were recorded by an automatic blood pressure monitor in a sitting position at rest. Participants self-assessed their puberty stage by viewing illustrations of different Tanner stages. Self-reported data on activity levels, alcohol consumption, and smoking status were also collected. If available, continuous glucose monitoring (CGM) data, the percentage of time that a participant spends within their target glycemic range (TIR) and hypoglycemia, were obtained through a 14-day CGM dataset.

The Composite Autonomic Symptom Score (COMPASS)-31 questionnaire was completed online by participants from their homes in the weeks prior to the test day. COMPASS-31 is a validated self-assessment instrument that includes six domains of autonomic symptoms: orthostatic, vasomotor, secretomotor, gastrointestinal, bladder, and pupillomotor [14]. It is suitable for widespread use in autonomic research and practice, that has been validated in younger individuals [15].

### Sudomotor function test: QSART

QSART [16], which evaluates sweat volume response after sudomotor sympathetic C-fiber activation with acetylcholine iontophoresis, was performed on the right side of the body at four locations; the forearm, proximal lower leg, distal leg, and foot using the WR TestWorks Q-Sweat quantitative sweat measurement system (WR Medical Electronics Co., Maplewood, MN, USA). An abnormal sweat response at the distal leg (QSART_distal_) was defined as a sweat response of less than the 5th percentile of the control group’s data. An abnormal response at the foot (QSART_foot_) was defined as reduced sweat response (< 5th percentile) and/or a length-dependent decrease, indicated by a sudomotor volume at the foot less than one-third that of the proximal sites [17].

### Sudomotor structural test: SGNFD

 Skin biopsies with a diameter of 4 mm were taken from the distal leg, 10 cm proximal to the lateral malleolus. Local anesthesia was applied before the procedure.

**Fig. 1:**
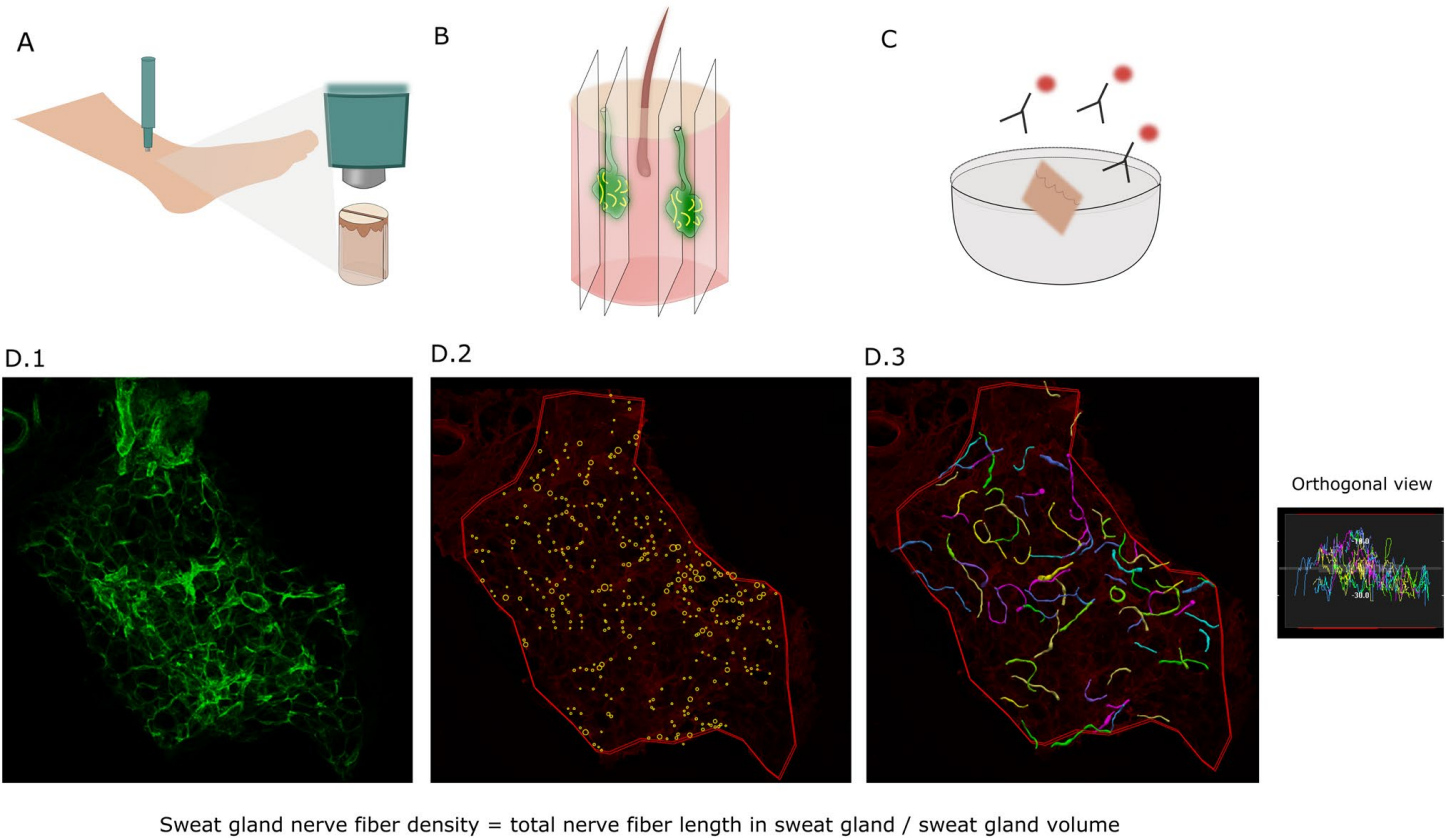
Illustration of the methods from collecting a skin sample to final analysis. **A**, **B**, and **C** are illustrations of the first steps in the method: **A** skin biopsy; **B** cutting in tissue sections (thickness 50 µm); **C** immunostaining. The images (**D.1**, **D.2**, and **D.3**) are from a healthy control subject and they show the step in analysis: **D.1** image of a sweat gland stained with collagen IV; **D.2** automatic setting of seeds (yellow dots) on PGP 9.5-stained nerves. Red outer line: contour of the sweat gland; **D.3** three-dimensional reconstruction of the nerves

The method of quantifying SGNFD has been previously described [9]. The description of the method has been updated with the current names of companies, software programs, and automatic settings, and is reused in the following section. Figure 1 illustrates the process.

#### Imaging

Approximately ten tissue sections were thoroughly searched for sweat glands (SGs). SGs were identified by the visualization of collagen IV staining of tubular basement membranes in association with protein gene product (PGP) 9.5 antibody signaling of nerve fibers. Six SGs per participant were imaged (3D Z stacks acquired at 1 µm intervals) with a ×10 objective, (LSM 800 Airyscan, Laser Scanning Confocal with Superresolution, Zeiss, Wetzlar, Germany) at the Department of Bioimaging, Aarhus University, Denmark.

 Images were deconvolved to reduce nonspecific background staining and increase resolution (Huygens Essentials version 18.04; Scientific Volume Imaging, Hilversum, Netherlands, http://svi.nl).

#### Sweat gland volume estimation

We used the contouring function in Neurolucida 360, a software platform for the reconstruction of neuronal morphology (NL360 version 2019.1.1, MBF Bioscience, Williston, VT, USA), to trace the region of the SG). A peripheral contour of the SG was drawn at the topmost Z-section and bottom Z-section of the image stack. Sweat gland volume (SGv) was defined as the area within the contours.

#### Morphometric 3D reconstruction

The image stained for nerve structures was downloaded in the same location as the image of SG.

Nerve fibers were reconstructed using the NeuroLucida 360 software program. We used the automated algorithm to trace nerve fibers from 3D (volumetric) Z-stacks. This method locates an initial point, places seeds in linear branched structures, and then exploits local image properties to trace the structures recursively in x, y, and z planes. Seeds were placed at 80% sensitivity and validated using the refine filter setting at level four. Additional modifications were made in edit mode to remove seeds placed outside SG structures, often related to nonspecific lipofuscin staining. After the automatic tracing of nerves, a manual editing process was applied to ensure that the stained structures were nerves. All images were analyzed while blinded, with an ID number that did not reveal whether they were from patients or controls.

Total nerve fiber length in SG (SGNFL) was quantified using the NeuroExplorer software program (MBF Bioscience), and SGNFD was calculated by dividing SGNFL by SGv.

An interrater coefficient (ICC) of 0.96 was found for the mean SGNFD (10 cases, 61 SG) when using the described method prior to the start of this study.

#### Data representation

For each participant, the mean, standard deviation (SD), minimum, median, and maximum values of SGNFL, SGv, and SGNFD were obtained from the analyzed SG per individual. When presenting the data for a group, calculations of mean, SD, 5th percentile, 50th percentile, and 95th percentile were performed for each SG parameter.

### Statistical analysis

All statistical analyses were conducted using the R software program (R Core Team 2022, Vienna, Austria). The Shapiro–Wilk test and Q–Q plot were used to test for the normality of all variables in Table 1. Descriptive data are presented as mean (SD) for continuous variables, median (range) for continuous variables with non-normal distribution, and number (%) for categorical variables. The participants were divided into two groups: one group with adolescents with T1D, and one group with healthy control subjects. Differences between groups were compared using Student’s *t* test for continuous variables with normal distribution, Wilcoxon rank-sum test for nonparametric continuous variables, and Fisher’s exact test for categorical variables. A *p* value of less than 0.05 was considered statistically significant, and we estimated that our sample size would comply with this. Bonferroni-correct *p* value was used in risk factor analysis to take into account the multiple testing. Pearson's product–moment correlation (cor.test function in R) and linear regression (lm function in R) was used for analyzing associations between parameters in each group.

**Table 1:** Characteristics of the study population

Variable	Control, *N* = 23^a^	Type 1 diabetes, *N* = 59^a^	*p* value^b^
Gender (female)	16 (70%)	29 (49%)	0.14
Age (years)	16.60 (15.40–18.20)	16.90 (15.00–18.90)	0.69
Diabetes duration (years)	0.0 (0.0–0.0)	8.5 (4.6–17.4)	** < 0.01**
HbA1c (mmol/mol [%])	33 (27–40) (5.2 (4.6–5.8))	60 (41–93) (7.6 (5.9–10.7))	** < 0.01**
BMI (kg/m^2^)	21.12 (16.90–30.40)	22.88 (17.63–29.61)	**0.03**
BMI-SDS	0.03 (-1.80 to – 1.68)	0.57 (– 2.30 to 1.85)	** < 0.01**
Height (cm)	174 (158–188)	172 (150–191)	0.97
Hip circumference (cm)	98 (65–112)	98 (76–114)	0.19
Waist circumference (cm)	74 (59–92)	75 (53–100)	0.53
Tanner (stage)			0.43
4	5 (22%)	19 (32%)	
5	18 (78%)	40 (68%)	
SBP (mmHg)	114 (98–130)	118 (68–147)	0.20
DBP (mmHg)	71 (59–89)	77 (55–96)	**0.03**
Pulse (BPM)	70 (55–99)	77 (48–106)	0.25
Retinopathy (yes)	0 (NA%)	3 (5.1%)	1.00
Nephropathy (yes)	0 (NA%)	2 (3.4%)	1.00
Cholesterol (mmol/L)	3.80 (2.80–5.10)	4.10 (3.00–6.40)	0.18
LDL (mmol/L)	2.10 (1.40–3.50)	2.10 (0.50–4.10)	1.00
HDL (mmol/L)	1.30 (0.68–2.20)	1.50 (0.97–3.70)	**0.05**
Triglycerides (mmol/L)	0.70 (0.30–1.10)	0.90 (0.30–3.80)	**0.01**
Alcohol (units/week)			**0.03**
0	1 (4.3%)	6 (10%)	
1–3	20 (87%)	28 (47%)	
4–7	2 (8.7%)	16 (27%)	
8–14	0 (0%)	5 (8.5%)	
> 15	0 (0%)	4 (6.8%)	
Smoking (status)			0.64
Never	18 (78%)	46 (78%)	
Previous	4 (17%)	6 (10%)	
Smoke	1 (4.3%)	6 (10%)	
NI	0 (0%)	1 (1.7%)	
Activity (hours/week)			0.16
0	0 (0%)	5 (8.5%)	
1–3	2 (8.7%)	14 (24%)	
4–7	8 (35%)	18 (31%)	
> 7	13 (57%)	22 (37%)	
HbA1c mean 5 years (mmol/mol [%])	NA	59 (40–95) (7.6 (5.8–10.8))	
Total daily insulin (units)	NA	59 (27–141)	
Time in range (%)	NA	54 (23–85)	
Basal insulin (units)	NA	28 (10–56)	
Time in hypoglycemia (%)	NA	5.0 (0.0–15.0)	

Missing data were excluded. *P* values in bold indicate a significant difference

*HbA1c* hemoglobin A1c, *BMI* body mass index, *SBP* systolic blood pressure, *DBP* diastolic blood pressure, *HDL* high-density lipoprotein, *LDL* low-density lipoprotein, *NI* not indicated

^a^Median (range) for continuous; *n* (%) for categorical

^b^Categorical variables, Fisher's exact test; continuous variable with normal distribution, Welch two-sample *t* test; continuous variable with non-normal distribution, Wilcoxon rank-sum test

Abnormality for diagnostic tests was defined as values below the 5th percentile compared with the normative data of the healthy included subjects.

The relative risk ratios were calculated using the 2×2 count data presented in a table, with the *epi.2by2* function in R. In the relative risk analysis, the participants were divided into subgroups within each variable as previously done in the population [12].

## Results

Sixty adolescents and 23 controls were enrolled in the study. None of the participants had a history of neuropathic symptoms. Twelve out of the 60 adolescents with T1D (20%) used pens to inject insulin, and the remaining 80% had an insulin pump. All used continuous glucose monitors (CGM) except for five participants. The median of self-monitoring of daily blood glucose frequency was 7 (range 1–25).

**Fig. 2:**
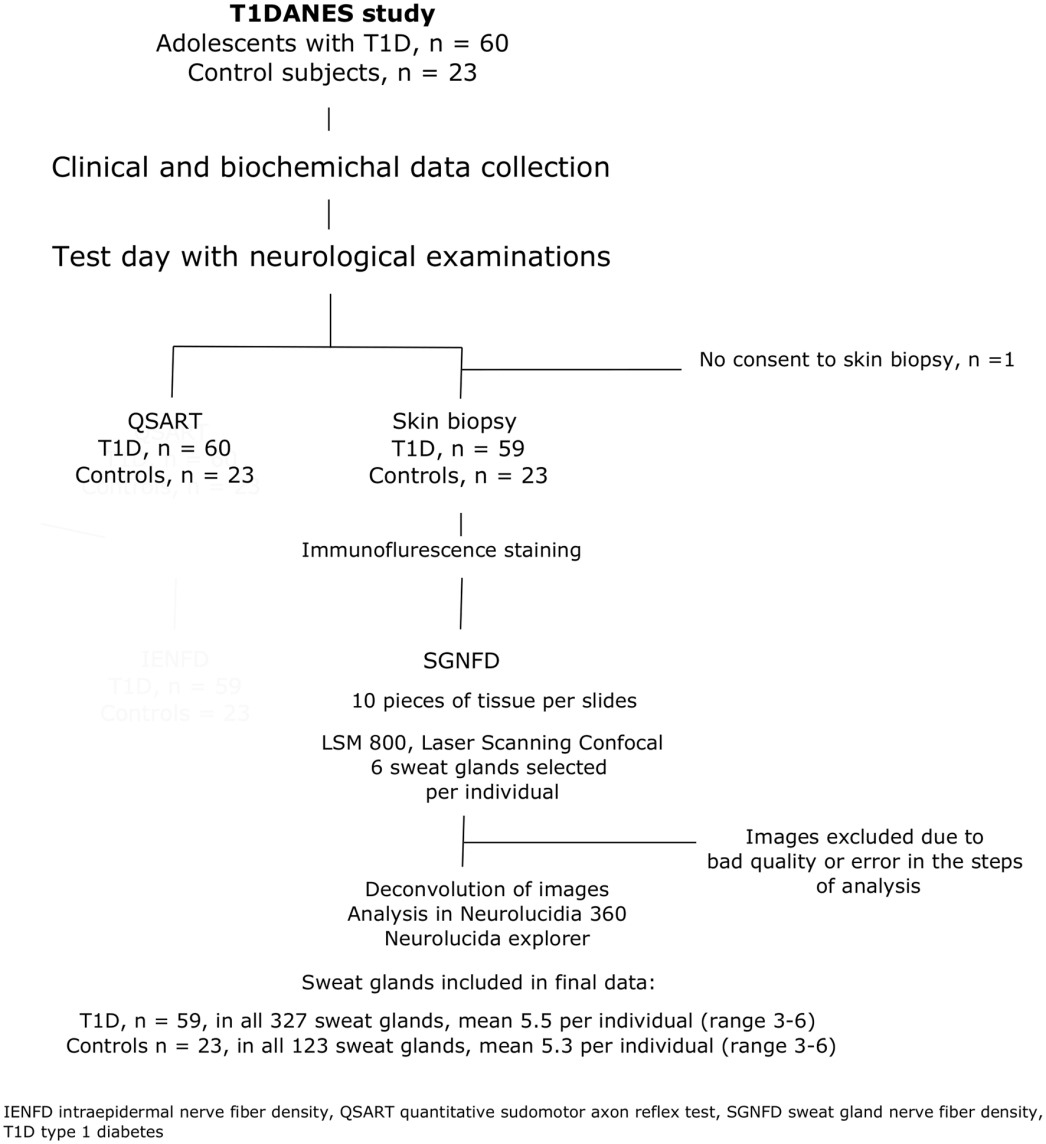
Flowchart of study population selection and data included in the final analysis

The selection process for the study population and the data available for final analysis are shown in Fig. 2, and the clinical characteristics of the participants are displayed in Table 1.

### Normative data of SGNFL, SGv, and SGNFD obtained from healthy control subjects

A total of 126 SG were analyzed, with a mean of 5.5 (SD 0.9, range 3–6) SGs per control subject.

The SGNFL_mean_ in the control group was 0.98 (SD 0.51) mm, SGv_mean_ was 0.314 (SD 0.132) × 10^3^ mm^3^, and SGNFD_mean_ was 3.03 (SD 1.07) m/mm^3^. The normative data for SGNFL, SGv, and SGNFD are shown in Appendix A.

There was no significant difference observed in the SGNFL and SGv parameters between males and females (SGNFL_mean_ *p* = 0.97; and SGv_mean_ *p* = 0.55). There was also no significant difference for SGNFD_mean_ (3.16 vs. 2.75 m/mm^3^, *p* = 0.37), but the SGNFD_maximum_ was higher in females than in males (5.33 vs. 3.90 m/mm^3^, *p* = 0.01). A higher SGNFL was significantly associated with higher SGv (*p* < 0.01) and higher SGNFD (*p* < 0.01). No association was observed between SGNFD_mean_ and SGv_mean_ (*p* = 0.20). Linear regression models applied to clinical factors showed that the ratio of waist circumference to height was positively correlated with SGNFL (*p* = 0.04), and higher age was associated with higher SGv (*p* < 0.01).

### Data on SGNFL, SGv, and SGNFD obtained in adolescents with type 1 diabetes

In total, 327 SG were analyzed, with a mean of 5.5 (SD 0.8, range 3–6) sweat glands per patient. In the T1D group, SGNFL_mean_ was 0.690 (SD 0.49) mm, SGv_mean_ was 0.277 (SD 0.11) × 10^3^ mm^3^, and SGNFD_mean_ was 2.43 (SD 1.00) m/mm^3^. All data on adolescents with T1D for SGNFL, SGv, and SGNFD are presented in Appendix B. A trend of higher SGNFL and SGv parameters in females than in males was observed, and this trend was significant for SGNFL_mean_ (*p* = 0.03), SGv_maximum_ (*p* < 0.01), and SGv_mean_ (*p* = 0.02). No significant sex differences in SGNFD parameters were found in the T1D group.

Higher SGNFL was significantly associated with higher SGv and higher SGNFD (*r* = 0.77; *r* = 0.78, and both *p* values < 0.01), and higher SGNFD was associated with higher SGv (*r* = 0.43, *p* < 0.01).

### Comparison between T1D and control group

Adolescents with T1D had a lower SGNFL_maximum_ (*p* < 0.01) and SGNFL_mean_ (*p* = 0.03) than control subjects, corresponding to a lowering of 29.6% in SGNFL_mean_.

The sweat gland volume (SGv) was similar in adolescents with T1D compared to control subjects (*p* = 0.24).

**Fig. 3:**
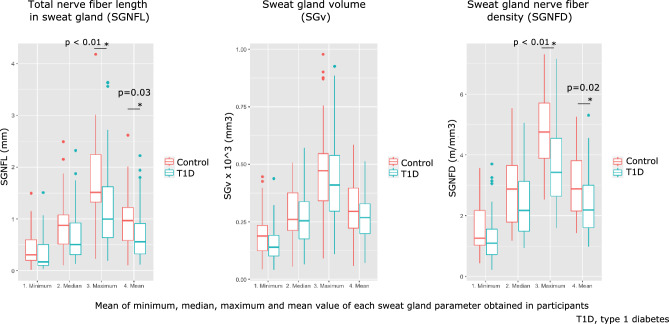
Sweat gland parameters in adolescents with type 1 diabetes and healthy control subjects

Adolescents with T1D had a lower SGNFD_maximum_ (*p* < 0.01) and SGNFD_mean_ (*p* = 0.02) compared to control subjects, corresponding to a lowering of 19.8% in SGNFD_mean_. These results are illustrated in Fig. 3.

### Association between sweat gland parameters and sweat response

Trends of positive associations between SGNFL and sweat response measured by QSART_distal leg_ (*r* = 0.24, *p* = 0.07) and SGv and QSART_distal leg_ (*r* = 0.22, *p* = 0.09) were observed in the T1D group but were not statistically significant. No association between SGNFD_mean_ and QSART _distal leg_ was found (*r* = 0.16, *p* = 0.21).

Some adolescents with T1D had only one affected QSART response, e.g., on the forearm (*n* = 5) or distal leg (*n* = 1). No correlations between other QSART placements (forearm, proximal leg, and foot) with SGNFD were found (data not shown, all *p* values < 0.05).

### Cases with abnormal SGNFD and QSART

In total, 34% (20 out of 59) of the adolescents with T1D had reduced SGNFD. In total, 20% (12 out of 59) had abnormal QSART: seven had reduced QSART_distal leg_, and 12 had abnormal QSART_foot_. When using the 5th percentile as a cutoff based on sex, three additional males showed affected QSART_distal leg_, and four additional males showed affected QSART_foot_. There was no change in the number of females with abnormal QSART_distal leg_ or QSART_foot_.

Table 2 provides an overview of cases with abnormal sudomotor tests, including the results of SGNFL, SGv, SGNFD, QSART, and reporting of symptoms in COMPASS-31_Sudomotor Items_.

**Table 2:** Clinical presentations in adolescents with type 1 diabetes with abnormal sudomotor tests

Affected sudomotor test	Sweat gland parameters: SGNFL, total nerve fiber length in sweat gland SGv, sweat gland volume SGNFD, sweat gland nerve fiber density	Sweat response obtained by quantitative sudomotor axon reflex test (QSART)	COMPASS-31_sudomotor ITEM 8_ A: I sweat much more than I used to B: I sweat somewhat more than I used to C: I have not noticed any changes in my sweating D: I sweat somewhat less than I used to E: I sweat much less than I used to
QSART_distal_ *n* = 7	1 case with low SGNFL, reduced SGv, and reduced SGNFD	Abnormal sweat response at distal leg	C, *n* = 1
	3 cases with low SGNFL and SGNFD		C, *n* = 3
	3 cases with normal SGNFL, SGv, SGNFD		C, *n* = 3
QSART_foot_ *n* = 12	3 cases with low/reduced SGNFL, reduced SGv, and reduced SGNFD	Reduced/absence of sweat response at foot and a length-dependent decrease at leg	B, *n* = 1 (dry mouth, *n* = 1); C, *n* = 2
	2 cases with low SGNFL and reduced SGNFD		C, *n* = 2
	7 cases with normal SG parameters		B, *n* = 1; C = 6 (dry eyes, *n* = 1)
SGNFD *n* = 20	3 cases with low/reduced NFL, reduced SGv, and reduced SGNFD	No response (foot), *n* = 2 Reduced response (foot and distal), *n* = 1	B, n = 1 (+ dry mouth); C, *n* = 2
	10 cases with low/reduced NFL and SGNFD	No response (foot and distal), *n* = 1 Reduced response (foot and distal), *n* = 1 Normal, *n* = 8	C, *n* = 10
	7 cases with only reduced SGNFD	Reduced (slightly below cutoff), *n* = 2 Normal, *n* = 5	B, *n* = 1; C, *n* = 6 (dry mouth, *n* = 1)

*QSART* quantitative sudomotor axon reflex test, *SG* sweat gland, *SGNFL* total nerve fiber length in sweat gland, *SGNFD* sweat gland nerve fiber density, *SGv* sweat gland volume, *COMPASS-31* composite autonomic symptom score 31. Reduced < 5th percentile cut-off. Low SGNFL < 0.35 mm

As part of a related study published elsewhere, neurological examination and nerve conduction studies were performed [12]. Only eight participants had abnormal bilateral findings on neurological examination, and of these, four had one affected SG parameter: one had reduced SGNFL, one had reduced SGv, and two had reduced SGNFD. The participant with reduced SGNFL had signs of both large- and small-fiber damage on neurological examination. There was no correlation between sweat gland parameters (SGv, SGNFL, SGNFD) and conduction velocity in the nerve conduction studies obtained in the other study [12] (data not shown, all *p* values > 0.05).

### Risk factors

Height, systolic blood pressure, total daily insulin dose, basal/total insulin dose, and time in hypoglycemia were positively correlated to sweat response, while low-density lipoprotein, and HbA1c (mean of the previous 5 years) were negatively correlated to sweat response (all *p* values < 0.05). However, only the analysis of height and basal/total insulin dose ratio was below the *p* value threshold criteria adjusted by Bonferroni correction (*p* value < 0.003). There were no correlations between HbA1c, diabetes duration, and TIR with sweat gland parameters (sweat response measured by QSART, SGNFL, SGv, and SGNFD). We found no other clinical variables associated with SG parameters than those mentioned above, even though younger age was associated with lower SGv (*p* = 0.03).

Dividing the participants into subgroups, the relative risk factors for reduced SGNFD included being older than 17 years (relative risk ratio (RR) 2.47 (1.26, 4.84)), cholesterol level ≥ 5 mmol/l (RR 2.09 (1.12, 3.91)), and having other microvascular complications (RR 32.4 (4.09, 256). The four adolescents with reduced SGNFD in combination with retinopathy or nephropathy had a diabetes duration between 5.6 and 9.9 years. The presence of poor metabolic control (> 9% (75 mmol/mol)), diabetes duration above 10 years, being female, and reduced level of physical activity did not increase the relative risk for abnormal sweat response or SGNFD (data not shown, all with nonsignificant RR ratio). Adolescents with a self-reported pubertal stage at Tanner 4 did not result in a higher relative risk for abnormal sweat response or reduced SGNFD compared to those with Tanner stage 5 (QSART RR 1.08 (0.37;3.15); SGNFD RR 0.65 (0.28;1.53)).

## Discussion

We report, to our knowledge for the first time, that adolescents with T1D had significantly reduced maximum and mean values of SGNFL and SGNFD compared to healthy control subjects. Additionally, to the best of our knowledge, this is the largest study of individuals with diabetes in which their sudomotor innervation has been quantified. A detailed morphological assessment of sweat glands is crucial for understanding morphological changes in autonomic structures such as sweat glands in diabetes. Thus, we evaluated not only the nerve fiber innervation in the sweat glands but also the length of the nerve fibers and the volume of the sweat glands.

The biological question of whether sweat glands shrink when they become dysfunctional is of interest. Loavenbruck et al. found a decrease in total SGv per biopsy in 10 cases with anhidrosis diagnosed with thermoregulatory sweat test compared to controls. They also found that the total nerve fiber length per biopsy volume decreased, but because of a greater change in SGv, SGNFD increased [18]. This contradicts our findings with similar SGv and a reduced SGNFL as the main explanation for the reduction of SGNFD, highlighting the importance of detailed morphological assessments of sweat glands, as the effect of different diseases on their structure and function may vary.

Our findings of lower mean values of SGNFL in the T1D group compared to controls support the notion of a reduction in the number of nerves, similar to findings when quantifying IENFD in people with diabetes [19]. The clinical impact of assessing only one affected SG parameter was of lesser significance; however, a combination of reduced SGv, SGNFL, and SGNFD was observed to have clinical consequences including either no sweat response or reduced sweat response.

**Fig. 4:**
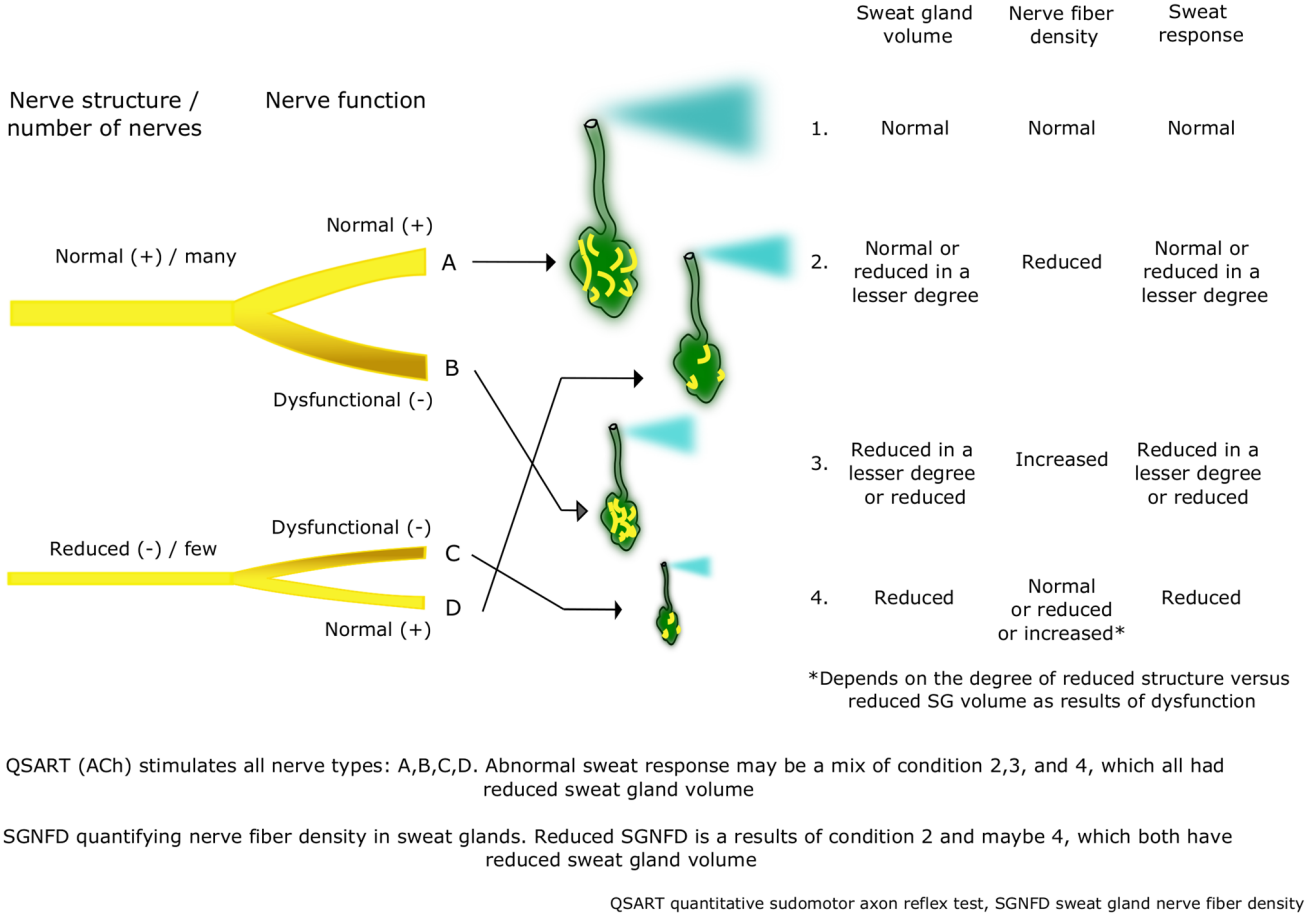
An explanatory model for different conditions of sudomotor innervation, sweat gland volume, and sweat response

We have illustrated four possible conditions that may explain the relationship between SG parameters (SGNFL, SGv, SGNFD) and sweat response in Fig. 4. Our possible explanation is that dysfunctional nerves may lead to a shrinkage of sweat glands, resulting in a reduced sweat response. A smaller number of nerves (lower SGNFL) may also impact sweat gland function but to a lesser extent. As illustrated in Fig. 4, four possible conditions exist: (1) a condition with a normal number of nerves with normal nerve function leading to normal SGv, SGNFD, and sweat response, (2) a condition with fewer nerves, but with normal nerve function, leading to a limited lowering of SGv and sweat response given a reduced SGNFD, (3) a condition with a normal number of nerves, but they are dysfunctional, leading to a shrinkage of sweat glands and a lower sweat response, but higher SGNFD, and (4) a condition with fewer nerves, which are also dysfunctional, leading to a pronounced shrinkage and low or no sweat response with normal, reduced, or increased SGNFD depending on the degree of reduction of SGNFL relative to SGv.

In our study, the majority of affected cases displayed sweat gland parameters consistent with condition 2 in Fig. 4: reduced SGNFD with hypothetically well-functioning nerves. This also explains why we found an association between lower SGNFL and lower sweat response.

A few of the patients in this study with abnormal SGNFD showed all three SG parameters (SGNFL, SGv, SGNFD) affected, similar to condition 4 in Fig. 4. Those cases exhibited the most severe reduction or absence of sweat response.

In theory, all patients could exhibit a combination of all conditions illustrated in Fig. 4. When conducting QSART on a patient, all nerves (A, B, C, D) are stimulated with acetylcholine, and an abnormal sweat response may be a mixture of conditions 2, 3, and 4, whereas abnormal SGNFD will only occur in conditions 2 and 4. The lack of association between QSART and SGNFD may be due to the fact that abnormal QSART also encompasses condition 3, leading to an even higher SGNFD. Additionally, acetylcholine in QSART does not stimulate adrenergic nerves, which are taken over if the parasympathetic system is damaged [20].

Two key points we want to emphasize if our explanatory model proves correct are that (1) reduced, normal, and increased SGNFD can all indicate an abnormal nerve status, depending on SGv, and (2) incorporating the quantification of sudomotor innervation into the QSART diagnostic method could help us distinguish between different conditions and evaluate both somatic and autonomic C-fibers. If the intra-individual variation in SGv, SGNFL, and SGNFD is low, patients may present a reduced mixture of conditions.

Our study found no association between SGNFD and age, although SGv increased with age. Considering that distal IENFD is known to decrease with advancing age [21], we suspect that our data may be best explained by the limited age range of our study population.

Our study found that most adolescents with T1D have measurable structural nerve changes and to a lesser extent reduced sweat response. Even fewer reported subjective changes in sweating. The idea that adolescents have preserved the ability to sweat in spite of structural changes in innervation seems plausible given their young age, fewer comorbidities, and fewer other confounding factors such as medications.

We found several lifestyle factors negatively associated with sweat gland innervation, including alcohol consumption, low-density lipoprotein, cholesterol, BMI, waist-to-height ratio, and smoking. Gender, BMI, and HbA1c have been previously described as factors that lower SG innervation in people with diabetes [8]. In addition, alterations in lipid metabolism and immuno-inflammatory factors are also known to be associated with diabetic neuropathy [22, 23]. It is known that abnormal lipoprotein levels can affect blood vessels supplying nerves and disrupt the lipid balance in the myelin sheath. Hyperlipidemia, particularly hypertriglyceridemia, is associated with the development and progression of neuropathy [24]. Also, excess glycated protein can contribute to nerve damage, forming advanced glycation end products (AGEs) that impair nerve function [25].

Our study showed that higher blood pressure correlates with higher sweat response, possibly due to increased activity of the sympathetic nervous system affecting both the blood vessels and SGs when the nerve function is intact. This could explain why blood pressure does not correlate with our structural nerve findings. Notably, none of our included adolescents in both groups were obese, which is of relevance as high blood pressure, obesity, and increased sweat response often coexist. Despite these facts, we still found that the waist-to-height ratio was positively correlated with SGNFL in the control group, but not in the T1D group. A higher waist-to-height ratio is a measure of central obesity, and whether the need for more sweating results in a higher SGNFL in healthy individuals with no risk of nerve damage remains to be investigated.

Despite the method being based on staining and automatic tracing, the technique of quantifying sudomotor innervation has limitations. Firstly, it requires a skin biopsy, which is an invasive procedure. In our study, the procedure was well-tolerated by adolescents when performed under local anesthesia. Additionally, deviations in the method steps can affect the final results, including the handling of the skin biopsy (fixative and storing), the staining protocol, the antibodies used, the choice of SG for analysis, the type of confocal microscope and its settings (magnification, image resolution, bit depth, gain settings), the program for removing background, and methods for tracing nerves. This highlights the importance of standardizing the entire process. We tried different automatic settings to minimize limitations before analyzing the included SG, and outlined optimized methodology in the methods section. Additionally, structures other than nerves that could lead to mis-tracing were excluded for the SG contour analysis. These structures include the contour of lipid droplets, fractionated or folded pieces of tissue, and arrector pili muscles, which have relatively dense autonomic innervation [26, 27]. It is important to note that the contour peripherally may overestimate the SGv, and using a ×10 objective may risk missing small nerve branches. The number of SGs, size of SGs, and density of innervation may vary depending on location, emphasizing the need for well-matched normative data. Additionally, it is important that the skin biopsy is taken deep enough to include the dermis.

Further research into normal physiological variations in SGv and sudomotor innervation is needed. Our study had more females than males in the control group, which can be considered a limitation. Furthermore, our small study population restricts us to adjust for risk factors; for example, sex differences are of importance. Healthy males tend to exhibit higher sweating rates than females [28], although the findings are not always uniform in all age groups [29]. The sex differences are primarily attributed to hormonal variations, body composition, and differences in metabolic heat production [30]. Park et al. showed that there is no difference in total activated sweat glands in males and females in their early 20s [31]; however, additionally, research is needed to establish clear conclusions. Our data showed that SGNFD_maximum_ was higher in healthy females than in healthy males, mainly caused by a nonsignificant lowering of SGv. Women tend to have more SGs but men's SGs are more active. Some studies have supported lower sweat output per SG in women than men [32]. We had no data on where the young women were in their menstrual cycle, which could be of relevance due to the impact of hormones on sweating [33]. Estrogen has a tendency to enhance heat dissipation responses, which can lead to lower body temperatures, whereas progesterone tends to promote higher body temperature, thereby influencing the autonomic mechanism of thermoregulation [34]. By periodically assessing resting sweat response, researchers can observe changes in sweat composition or rate that may occur naturally or due to various factors, such as aging, change in hormone profile, and disease progression. However, when we are specifically interested in a functional investigation of the adrenergic nerves, QSART might be superior, as the nerves are stimulated.

Another limitation of our study is that we had no information about diet, because nutrition is known to impact sweating [35]. However, on the test day, they received the same standard meal as part of another test [13], which might have minimized this factor. Finally, the test only evaluates the SG innervation at the exact spot where the skin biopsy was taken. This spot was not exactly the same location as where the distal QSART capsule was placed.

Despite these limitations and critical evaluation of the methods, our study demonstrated that quantifying sweat gland innervation in a 3D manner is possible. The analysis of IENFD and SGNFD can be performed using the same skin biopsy, and many laboratories already have established protocols and equipment. Altogether, the quantification of SG parameters may complement the results obtained from QSART. However, it is important to note that the test requires proper handling of the skin biopsy and trained personnel for analysis, and it may not be available in many places. The number of selected SGs per individual can be debated, but, for clinical use, approximately 5–6 SGs per individual appears to be sufficient, as shown in other studies of IENFD [36]. Currently, there is no clinical significance in routinely performing skin biopsies and quantifying sudomotor innervation in all adolescents with diabetes. The study has provided new insights into the field. However, individuals with autonomic symptoms are considered to benefit from general autonomic testing including QSART [37].

The strength of this study lies in its large population of 60 adolescents with T1D, whose clinical parameters were well documented. Clinical tests were standardized by the same trained healthcare professionals using the same equipment on all participants (both T1D and control subjects). The entire SGNFD analysis process, from start to finish, was performed by a single person to minimize the risk of inconsistent processing due to multiple observers.

In conclusion, SGNFD and QSART were found to be useful in evaluating sudomotor innervation and function in adolescents. Results showed that adolescents with T1D had reduced SGNFL and SGNFD compared to controls. Both SGNFL and SGNFD were found to be associated with SGv, which was identified as an important factor in the association with sweat response. Notably, reduced, normal, and increased SGNFD can all be a sign of neuropathy, depending on the SGv, if our explanatory model is proven true. The addition of more diagnostic tests may be desirable in mapping the distribution of neuropathy, differentiating between structural and functional nerve damage, and further advancing our understanding of the underlying causes of sudomotor failure. However, further research is needed to understand its clinical relevance, before it can be recommended to be incorporated in clinical practice.

### Supplementary Information

Below is the link to the electronic supplementary material.

Supplementary file1 (DOCX 30 kb)

Supplementary file2 (DOCX 30 kb)

## Data Availability

The datasets generated during and/or analyzed during the current study are not publicly available due to the General Data Protection Regulation. However, an anonymized version of the datasets can be obtained from the corresponding author upon reasonable request and acceptance from the Legal Office, Central Denmark Region.

## Abbreviations

QSART: Quantitative sudomotor axon reflex tests
SG: Sweat gland
SGNFL: Total nerve fiber length in the sweat gland
SGv: Sweat gland volume
SGNFD: Sweat gland nerve fiber density
T1D: Type 1 diabetes

## References

[1] Rasmussen VF, Jensen TS, Tankisi H (2021). Large fibre, small fibre and autonomic neuropathy in adolescents with type 1 diabetes: a systematic review. J Diabetes Complicat.

[2] Franceschi R, Mozzillo E, Di Candia F (2022). A systematic review of the prevalence, risk factors and screening tools for autonomic and diabetic peripheral neuropathy in children, adolescents and young adults with type 1 diabetes. Acta Diabetol.

[3] Freeman R (2014). Diabetic autonomic neuropathy. Handb Clin Neurol.

[4] Terkelsen AJ, Karlsson P, Lauria G, Freeman R, Finnerup NB, Jensen TS (2017). The diagnostic challenge of small fibre neuropathy: clinical presentations, evaluations, and causes. Lancet Neurol.

[5] Gibbons CH, Illigens BM, Wang N, Freeman R (2010). Quantification of sudomotor innervation: a comparison of three methods. Muscle Nerve.

[6] Gibbons CH, Illigens BM, Wang N, Freeman R (2009). Quantification of sweat gland innervation: a clinical-pathologic correlation. Neurology.

[7] Luo KR, Chao CC, Chen YT (2011). Quantitation of sudomotor innervation in skin biopsies of patients with diabetic neuropathy. J Neuropathol Exp Neurol.

[8] Liu Y, Billiet J, Ebenezer GJ (2015). Factors influencing sweat gland innervation in diabetes. Neurology.

[9] Minota K, Schmeichel AM, Gehrking JA, Mandrekar JN, Low PA, Singer W (2019). Refined quantitation of sweat gland innervation. J Neuropathol Exp Neurol.

[10] Low PA (2003). Testing the autonomic nervous system. Semin Neurol.

[11] Libman I, Haynes A, Lyons S (2022). ISPAD clinical practice consensus guidelines 2022: definition, epidemiology, and classification of diabetes in children and adolescents. Pediatr Diabetes.

[12] Rasmussen VF, Thrysøe M, Nyengaard JR (2023). Neuropathy in adolescents with type 1 diabetes: confirmatory diagnostic tests, bedside tests, and risk factors. Diabetes Res Clin Pract.

[13] Rasmussen VF, Thrysøe M, Karlsson P (2023). Early gastrointestinal neuropathy assessed by wireless motility capsules in adolescents with type 1 diabetes. J Clin Med.

[14] Brinth L, Pors K, Mehlsn J, Sletten DM, Terkelsen AJ, Singer W (2021). Translation and linguistic validation of the composite autonomic symptom score COMPASS 31 in Danish. Dan Med J.

[15] Sletten DM, Suarez GA, Low PA, Mandrekar J, Singer W (2012). COMPASS 31: a refined and abbreviated composite autonomic symptom score. Mayo Clin Proc.

[16] Illigens BM, Gibbons CH (2009). Sweat testing to evaluate autonomic function. Clin Auton Res.

[17] Cheshire WP, Freeman R, Gibbons CH (2021). Electrodiagnostic assessment of the autonomic nervous system: a consensus statement endorsed by the American autonomic society, American academy of neurology, and the international federation of clinical neurophysiology. Clin Neurophysiol.

[18] Loavenbruck A, Wendelschaefer-Crabbe G, Sandroni P, Kennedy WR (2014). Quantification of sweat gland volume and innervation in neuropathy: correlation with thermoregulatory sweat testing. Muscle Nerve.

[19] Karlsson P, Hincker AM, Jensen TS, Freeman R, Haroutounian S (2019). Structural, functional, and symptom relations in painful distal symmetric polyneuropathies: a systematic review. Pain.

[20] Singer W, Spies JM, McArthur J (2004). Prospective evaluation of somatic and autonomic small fibers in selected autonomic neuropathies. Neurology.

[21] Umapathi T, Tan WL, Tan NC, Chan YH (2006). Determinants of epidermal nerve fiber density in normal individuals. Muscle Nerve.

[22] Rumora AE, Guo K, Alakwaa FM (2021). Plasma lipid metabolites associate with diabetic polyneuropathy in a cohort with type 2 diabetes. Ann Clin Transl Neurol.

[23] Xue T, Zhang X, Xing Y (2021). Advances about immunoinflammatory pathogenesis and treatment in diabetic peripheral neuropathy. Front Pharmacol.

[24] Iqbal Z, Bashir B, Ferdousi M (2021). Lipids and peripheral neuropathy. Curr Opin Lipidol.

[25] Papachristou S, Pafili K, Papanas N (2021). Skin AGEs and diabetic neuropathy. BMC Endocr Disord.

[26] Siepmann T, Gibbons CH, Illigens BM, Lafo JA, Brown CM, Freeman R (2012). Quantitative pilomotor axon reflex test: a novel test of pilomotor function. Arch Neurol.

[27] Nolano M, Provitera V, Caporaso G, Stancanelli A, Vitale DF, Santoro L (2010). Quantification of pilomotor nerves: a new tool to evaluate autonomic involvement in diabetes. Neurology.

[28] Low PA, Denq J-C, Opfer-Gehrking TL, Dyck PJ, O'Brien PC, Slezak JM (1997). Effect of age and gender on sudomotor and cardiovagal function and blood pressure response to tilt in normal subjects. Muscle Nerve.

[29] Lee JB, Lee IH, Shin YO, Min YK, Yang HM (2014). Age- and sex-related differences in sudomotor function evaluated by the quantitative sudomotor axon reflex test (QSART) in healthy humans. Clin Exp Pharmacol Physiol.

[30] Wickham KA, McCarthy DG, Spriet LL, Cheung SS (2021). Sex differences in the physiological responses to exercise-induced dehydration: consequences and mechanisms. J Appl Physiol (1985).

[31] Park T-H, Lee J-B, Lee H-J, Yun B (2020). Sex-related differences in sudomotor function in healthy early twenties focused on activated sweat gland density. Chin J Physiol.

[32] Baker LB (2019). Physiology of sweat gland function: the roles of sweating and sweat composition in human health. Temperature (Austin).

[33] Deecher DC, Dorries K (2007). Understanding the pathophysiology of vasomotor symptoms (hot flushes and night sweats) that occur in perimenopause, menopause, and postmenopause life stages. Arch Womens Ment Health.

[34] Charkoudian N, Stachenfeld N (2016). Sex hormone effects on autonomic mechanisms of thermoregulation in humans. Auton Neurosci.

[35] Baker LB, Wolfe AS (2020). Physiological mechanisms determining eccrine sweat composition. Eur J Appl Physiol.

[36] Engelstad JK, Taylor SW, Witt LV (2012). Epidermal nerve fibers: confidence intervals and continuous measures with nerve conduction. Neurology.

[37] Low PA (1993). Composite autonomic scoring scale for laboratory quantification of generalized autonomic failure. Mayo Clin Proc.

