# Supplementary material for: Sweat gland nerve fiber density and association with sudomotor function, symptoms, and risk factors in adolescents with type 1 diabetes
Source: Clin Auton Res. 2023 Sep 8;33(6):691–703. doi: 10.1007/s10286-023-00973-7 (PMC10751258; doi:10.1007/s10286-023-00973-7)
Supplement: Supplementary file 1 — Supplementary file1 (DOCX 30 kb) [file 10286_2023_973_MOESM1_ESM.docx]

**Appendix A.**

**Data obtained from healthy control subjects**

Adolescents, n = 23

Female/male 16/7

Mean age 16.7 yrs. (SD 0.7), range 15.4-18.2 yrs.

**Sweat gland nerve fiber density**

126 analyzed sweat glands (SG).

Number of SG analyzed per participants mean 5.5 (SD 0.8), range 3-6

| Measure | Number | Mean | SD | 5th percentile | 50th percentile | 95th percentile |
| --- | --- | --- | --- | --- | --- | --- |
| Total nerve fiber length (mm) | | | | | | |
| NFL minimum | 23 | 0.43 | 0.36 | 0.04 | 0.31 | 1.12 |
| NFL median | 23 | 0.92 | 0.61 | 0.12 | 0.88 | 2.12 |
| NFL maximum | 23 | 1.79 | 0.85 | 0.67 | 0.15 | 3.00 |
| NFL mean | 23 | 0.98 | 0.51 | 0.13 | 0.97 | 2.00 |
| Sweat gland volume (x 10^3 mm3) | | | | | | |
| SG volume minimum | 23 | 0.199 | 0.116 | 0.047 | 0.188 | 0.423 |
| SG volume median | 23 | 0.286 | 0.113 | 0.119 | 0.260 | 0.452 |
| SG volume maximum | 23 | 0.495 | 0.232 | 0.225 | 0.472 | 0.899 |
| SG volume mean | 23 | 0.314 | 0.131 | 0.137 | 0.295 | 0.555 |
| Sweat gland nerve fiber density (m/mm3) | | | | | | |
| SGNFD minimum | 23 | 1.61 | 0.91 | 0.71 | 1.26 | 3.43 |
| SGNFD median | 23 | 2.91 | 1.29 | 1.30 | 2.88 | 5.17 |
| SGNFD maximum | 23 | 4.89 | 1.34 | 2.97 | 4.75 | 7.09 |
| SGNFD mean | 23 | 3.03 | 1.04 | 1.80 | 2.88 | 5.01 |

NFL, total nerve fiber length; SG volume, sweat gland volume; SGNDF, sweat gland nerve fiber density

**Sweat gland nerve fiber density**

Controls, female

87 analyzed sweat glands (SG).

Number of SG analyzed per participants mean 5.4 (SD 0.6), range 4-6

| Measure | Number | Mean | SD | 5th percentile | 50th percentile | 95th percentile |
| --- | --- | --- | --- | --- | --- | --- |
| Total nerve fiber length (mm) | | | | | | |
| NFL minimum | 16 | 0.44 | 0.39 | 0.03 | 0.30 | 1.02 |
| NFL median | 16 | 0.91 | 0.65 | 0.22 | 0.88 | 2.24 |
| NFL maximum | 16 | 1.77 | 0.86 | 0.89 | 1.46 | 3.00 |
| NFL mean | 16 | 0.97 | 0.64 | 0.20 | 0.97 | 2.16 |
| Sweat gland volume (x 10^3 mm3) | | | | | | |
| SG volume minimum | 16 | 0.190 | 0.107 | 0.062 | 0.176 | 0.410 |
| SG volume median | 16 | 0.277 | 0.109 | 0.159 | 0.247 | 0.461 |
| SG volume maximum | 16 | 0.478 | 0.221 | 0.228 | 0.429 | 0.920 |
| SG volume mean | 16 | 0.300 | 0.117 | 0.172 | 0.282 | 0.515 |
| Sweat gland nerve fiber density (m/mm3) | | | | | | |
| SGNFD minimum | 16 | 1.55 | 0.89 | 0.63 | 1.22 | 3.15 |
| SGNFD median | 16 | 3.09 | 1.39 | 1.36 | 3.15 | 5.30 |
| SGNFD maximum | 16 | 5.33 | 1.33 | 3.21 | 5.60 | 7.17 |
| SGNFD mean | 16 | 3.16 | 1.14 | 1.70 | 2.91 | 5.12 |

**Sweat gland nerve fiber density**

Controls, male

39 analyzed sweat glands (SG).

Number of SG analyzed per participants mean 5.6 (SD 1.1), range 3-6

| Measure | Number | Mean | SD | 5th percentile | 50th percentile | 95th percentile |
| --- | --- | --- | --- | --- | --- | --- |
| Total nerve fiber length (mm) | | | | | | |
| NFL minimum | 7 | 0.42 | 0.36 | 0.07 | 0.35 | 0.98 |
| NFL median | 7 | 0.94 | 0.60 | 0.25 | 0.88 | 1.78 |
| NFL maximum | 7 | 1.83 | 0.97 | 0.40 | 2.00 | 2.83 |
| NFL mean | 7 | 1.00 | 0.58 | 0.25 | 1.18 | 1.72 |
| Sweat gland volume (x 10^3 mm3) | | | | | | |
| SG volume minimum | 7 | 0.222 | 0.148 | 0.059 | 0.188 | 0.404 |
| SG volume median | 7 | 0.308 | 0.135 | 0.110 | 0.374 | 0.435 |
| SG volume maximum | 7 | 0.534 | 0.285 | 0.152 | 0.524 | 0.875 |
| SG volume mean | 7 | 0.344 | 0.173 | 0.106 | 0.366 | 0.549 |
| Sweat gland nerve fiber density (m/mm3) | | | | | | |
| SGNFD minimum | 7 | 1.75 | 1.08 | 0.86 | 1.13 | 3.36 |
| SGNFD median | 7 | 2.49 | 1.14 | 1.45 | 2.13 | 4.25 |
| SGNFD maximum | 7 | 3.90 | 0.86 | 2.77 | 4.06 | 4.90 |
| SGNFD mean | 7 | 2.75 | 0.87 | 1.99 | 2.33 | 4.02 |
