# Supplementary material for: Sweat gland nerve fiber density and association with sudomotor function, symptoms, and risk factors in adolescents with type 1 diabetes
Source: Clin Auton Res. 2023 Sep 8;33(6):691–703. doi: 10.1007/s10286-023-00973-7 (PMC10751258; doi:10.1007/s10286-023-00973-7)
Supplement: Supplementary file 2 — Supplementary file2 (DOCX 30 kb) [file 10286_2023_973_MOESM2_ESM.docx]

**Appendix B.**

**Data obtained from adolescents with type 1 diabetes (T1D)**

Adolescents, n = 59

Female/male 29/30

Mean age 16.9 yrs (SD 1.13), range 15.0-18.95 yrs.

**Sweat gland nerve fiber density**

327 analyzed sweat glands (SG).

Number of SG analyzed per participants was 5.5 (SD 0.8), range 3-6

| Measure | Number | Mean | SD | 5th percentile | 50th percentile | 95th percentile |
| --- | --- | --- | --- | --- | --- | --- |
| Total nerve fiber length (mm) | | | | | | |
| NFL minimum | 59 | 0.32 | 0.32 | 0.05 | 0.17 | 0.89 |
| NFL median | 59 | 0.67 | 0.49 | 0.16 | 0.50 | 1.56 |
| NFL maximum | 59 | 1.20 | 0.80 | 0.35 | 1.00 | 2.80 |
| NFL mean | 59 | 0.69 | 0.49 | 0.18 | 0.56 | 1.79 |
| Sweat gland volume (x 10^3 mm3) | | | | | | |
| SG volume minimum | 59 | 0.156 | 0.080 | 0.063 | 0.139 | 0.300 |
| SG volume median | 59 | 0.271 | 0.118 | 0.107 | 0.254 | 0.497 |
| SG volume maximum | 59 | 0.436 | 0.179 | 0.188 | 0.409 | 0.847 |
| SG volume mean | 59 | 0.277 | 0.109 | 0.120 | 0.268 | 0.502 |
| Sweat gland nerve fiber density (m/mm3) | | | | | | |
| SGNFD minimum | 59 | 1.26 | 0.79 | 0.29 | 1.10 | 3.06 |
| SGNFD median | 59 | 2.34 | 1.11 | 1.12 | 2.17 | 4.39 |
| SGNFD maximum | 59 | 3.78 | 1.42 | 2.02 | 3.43 | 6.46 |
| SGNFD mean | 59 | 2.43 | 1.00 | 1.18 | 2.19 | 4.29 |

NFL, total nerve fiber length; SG volume, sweat gland volume; SGNDF, sweat gland nerve fiber density

**Sweat gland nerve fiber density**

T1D, female

156 analyzed sweat glands (SG).

Number of SG analyzed per participants was 5.4 (SD 0.95), range 2-6

| Measure | Number | Mean | SD | 5th percentile | 50th percentile | 95th percentile |
| --- | --- | --- | --- | --- | --- | --- |
| Total nerve fiber length (mm) | | | | | | |
| NFL minimum | 59 | 0.31 | 0.27 | 0.05 | 0.20 | 0.79 |
| NFL median | 59 | 0.76 | 0.50 | 0.23 | 0.67 | 1.67 |
| NFL maximum | 59 | 1.39 | 0.84 | 0.46 | 1.09 | 3.23 |
| NFL mean | 59 | 0.78 | 0.47 | 0.29 | 0.64 | 1.79 |
| Sweat gland volume (x 10^3 mm3) | | | | | | |
| SG volume minimum | 59 | 0.155 | 0.065 | 0.071 | 0.150 | 0.280 |
| SG volume median | 59 | 0.298 | 0.099 | 0.168 | 0.286 | 0.477 |
| SG volume maximum | 59 | 0.588 | 0.155 | 0.283 | 0.451 | 0.772 |
| SG volume mean | 59 | 0.301 | 0.086 | 0.178 | 0.296 | 0.451 |
| Sweat gland nerve fiber density (m/mm3) | | | | | | |
| SGNFD minimum | 59 | 1.17 | 0.76 | 0.24 | 1.04 | 2.38 |
| SGNFD median | 59 | 2.36 | 1.08 | 1.12 | 2.17 | 4.18 |
| SGNFD maximum | 59 | 3.84 | 1.23 | 2.21 | 3.98 | 5.89 |
| SGNFD mean | 59 | 2.44 | 0.90 | 1.39 | 2.24 | 3.85 |

**Sweat gland nerve fiber density**

T1D, male

170 analyzed sweat glands (SG).

Number of SG analyzed per participants was 5.7 (SD 0.7), range 4-6

| Measure | Number | Mean | SD | 5th percentile | 50th percentile | 95th percentile |
| --- | --- | --- | --- | --- | --- | --- |
| Total nerve fiber length (mm) | | | | | | |
| NFL minimum | 59 | 0.33 | 0.37 | 0.07 | 0.15 | 1.00 |
| NFL median | 59 | 0.59 | 0.48 | 0.15 | 0.38 | 1.53 |
| NFL maximum | 59 | 1.02 | 0.75 | 0.23 | 0.79 | 2.05 |
| NFL mean | 59 | 0.60 | 0.51 | 0.15 | 0.43 | 1.52 |
| Sweat gland volume (x 10^3 mm3) | | | | | | |
| SG volume minimum | 59 | 0.156 | 0.095 | 0.058 | 0.126 | 0.311 |
| SG volume median | 59 | 0.244 | 0.131 | 0.092 | 0.204 | 0.486 |
| SG volume maximum | 59 | 0.385 | 0.190 | 0.162 | 0.372 | 0.773 |
| SG volume mean | 59 | 0.253 | 0.125 | 0.112 | 0.221 | 0.502 |
| Sweat gland nerve fiber density (m/mm3) | | | | | | |
| SGNFD minimum | 59 | 1.32 | 0.84 | 0.40 | 1.12 | 3.10 |
| SGNFD median | 59 | 2.32 | 1.17 | 1.17 | 2.06 | 4.64 |
| SGNFD maximum | 59 | 3.71 | 1.62 | 1.93 | 3.20 | 6.67 |
| SGNFD mean | 59 | 2.41 | 1.13 | 1.17 | 2.13 | 4.45 |
